# Supplementary material for: Nonlinear reconfiguration of network edges, topology and information content during an artificial learning task
Source: Brain Inform. 2021 Dec 2;8(1):26. doi: 10.1186/s40708-021-00147-z (PMC8639979; doi:10.1186/s40708-021-00147-z)
Supplement: Supplementary file 1 — Additional file 1: Figure S1. Accuracy in eMNIST dataset. Figure S2. Correlation between topology and PC1 eigenvector. Figure S3. Classification performance was aligned with the low-dimensional embedding space. Figure S4. Dimensionality of Hidden Layers across training. [file 40708_2021_147_MOESM1_ESM.docx]

**Additional Figures**

**Fig S1.** **Accuracy in eMNIST dataset.** A) the increase in accuracy over training was conceptually equivalent to the MNIST dataset, but note that the classification accuracy was lower to begin with, due to the higher complexity of the training set (i.e., 26 letters > 10 digits); B) similar topological epochs were observed in the eMNIST dataset (Early – blue; Middle – green and Late – purple); C) I*_H_*  = MI(node,class) for *HL1* (blue) was higher than MI*_HL2_* (orange) in the Early phase, they both increased in the Middle phase, and in the Late phase, MI*_HL2_* continued to increase whereas MIHL1 diminished; D) strong positive correlation between IP and the change in edge strength over the Early phase (r = 0.829, p*_PERM_* = 0).

**Fig S2.** **Correlation between topology and PC_1_ eigenvector.** Relationship between MZ (blue) and PC (orange) for first layer in MNIST dataset across all epochs.

**Fig S3.** **Classification performance was aligned with the low-dimensional embedding space.** Results depict the difference in eigenvector embeddings across the first 100 eigenmodes. Green: FDR p < 0.05; Red: FDR p >= 0.05.

**
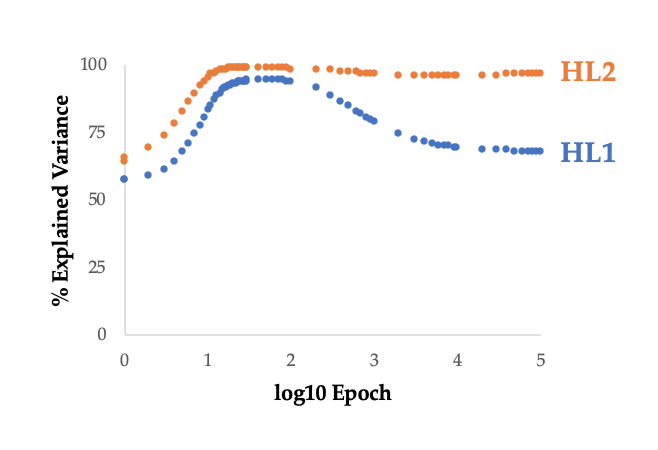
**

**Fig S4.** **Dimensionality of Hidden Layers across training.** Each point represents the sum of the % explained variance of the top 10 principal components fit on HL1 (blue) and HL2 (orange) within each training epoch.
